# Application of Nanocellulose-Based Aerogels in Bone Tissue Engineering: Current Trends and Outlooks

**DOI:** 10.3390/polym15102323

**Published:** 2023-05-16

**Authors:** Yaoguang Zhang, Shengjun Jiang, Dongdong Xu, Zubing Li, Jie Guo, Zhi Li, Gu Cheng

**Affiliations:** 1 The State Key Laboratory Breeding Base of Basic Science of Stomatology (Hubei-MOST), Key Laboratory of Oral Biomedicine Ministry of Education, School and Hospital of Stomatology, Wuhan University, Wuhan 430079, Chinalizubing@whu.edu.cn (Z.L.); 2 Department of Orthodontics, School and Hospital of Stomatology, Cheeloo College of Medicine, Shandong University, Shandong Key Laboratory of Oral Tissue Regeneration & Shandong Engineering Laboratory for Dental Materials and Oral Tissue Regeneration & Shandong Provincial Clinical Research Center for Oral Diseases, Jinan 250012, China; 3 Department of Stomatology, Renmin Hospital of Wuhan University, Wuhan 430079, China; 4 School and Hospital of Stomatology, Wenzhou Medical University, Wenzhou 325015, China

**Keywords:** nanocellulose, aerogel, bone tissue engineering, bone defect

## Abstract

The complex or compromised bone defects caused by osteomyelitis, malignant tumors, metastatic tumors, skeletal abnormalities, and systemic diseases are difficult to be self-repaired, leading to a non-union fracture. With the increasing demands of bone transplantation, more and more attention has been paid to artificial bone substitutes. As biopolymer-based aerogel materials, nanocellulose aerogels have been widely utilized in bone tissue engineering. More importantly, nanocellulose aerogels not only mimic the structure of the extracellular matrix but could also deliver drugs and bioactive molecules to promote tissue healing and growth. Here, we reviewed the most recent literature about nanocellulose-based aerogels, summarized the preparation, modification, composite fabrication, and applications of nanocellulose-based aerogels in bone tissue engineering, as well as giving special focus to the current limitations and future opportunities of nanocellulose aerogels for bone tissue engineering.

## 1. Introduction

As a common clinical disease, large-sized bone defects caused by trauma, infection, tumor, surgical debridement of osteomyelitis, and congenital diseases destroy the structural integrity of bone and have caused an urgent demand for highly effective bone substitutes in the coming years [1,2,3,4,5]. Strategies for repairing bone defects include distraction osteogenesis, autologous bone transplantation, allogeneic bone graft, and artificial grafts [2]. Currently, the gold standard approach is autologous graft transplantation, which has been widely used in clinical practice due to its advantages of non-immunogenic, histocompatibility, and excellent osteogenic ability [6]. However, the limited sources and secondary injury in the donor site have limited the usage of autologous bone grafts [5,6,7]. The application of distraction osteogenesis has also been hindered by a low success rate, long healing period, and narrow range of application [8]. Although allogeneic bone grafts partially overcome the above-mentioned drawbacks of autologous bone graft and distraction osteogenesis, they have also faced the risks of immune rejection, transplant site infection, and the poor qualities of the generated bone tissues [9,10,11].

Bone tissue engineering (BTE), which is based on the principles of biology and engineering, has been widely utilized in recent years to construct a substitute to repair and improve the function of bone [12]. This process is to seed enough seeding cells onto the artificial scaffolds to construct tissue-engineered compounds in vitro and then to regenerate the newly formed tissues in vivo. Therefore, successful BTE depends on enough undifferentiated cells, biomimetic scaffolds, and signaling molecules that induce cell differentiation [13], and the tissue-engineered scaffolds are expected to replace other existing bone grafts due to evident advantages such as unlimited supply and controllable physiochemical or mechanical properties [2,14,15,16].

In recent years, higher requirements for tissue-engineered biomaterials have promoted the design and development of double-pore (major pores with sizes ranging from 50 to 150 μm and minor pores with a size smaller than 20 μm) biomaterials. Compared with the traditional fully random porous structure, the double-pore scaffold system has significant advantages in BTE [17]. As an excellent double-pore scaffold, the minor pores (20–50 μm) of aerogels and their connectivity in 3D nanostructures facilitate cell attachment, and additionally, major pores (>50 μm) provide nutrient and oxygen channels to cells [18,19,20]. The controllable porous structure and designable flexibility and shape memory of aerogels have significant advantages in repairing bone defects, especially for those with irregular shapes [21].

Traditional aerogels are generally prepared based on inorganic components such as silica and metal oxide, while the mechanical brittleness of these inorganic aerogels severely limits their application in BTE [22]. The new generation of aerogels is mainly based on polysaccharide organic aerogels that appeared in the early 21st century. Compared with silica aerogels, the breaking of polysaccharide aerogels under compression is relatively difficult [23]. Among them, nanocellulose-based aerogels exhibited flexibility, repeatability, degradability, and excellent biocompatibility [24].

Cellulose aerogel is known as “the third-generation aerogel material” and has a great potential for BTE [25,26,27,28]. Recent studies have frequently focused on nanocellulose-based materials for the fabrication of degradable scaffolds and tissue regeneration [1,27,29]. However, the hydrophilicity, poor mechanical properties, and poor osteoconductive properties of nanocellulose aerogels restrict their applications in the field of BTE [30]. Notably, the modification and composition of the preparation procedures of nanocellulose aerogels can overcome these drawbacks to a certain degree [31]. Nanocellulose aerogels have special advantages in promoting tissue healing, and their composite has huge potential in manufacturing biomimetic scaffolds for tissue engineering [27,32]. In general, nanocellulose aerogels could be utilized alone and combined with other components, active molecules, and seed cells in various sources for BTE [33,34,35,36]. In this paper, we summarized the preparation and modification methods of nanocellulose aerogels, discussed the advantages of nanocellulose aerogels in drug loading and biomimetic manufacturing, and, finally, put forward their remaining limitations and future development.

## 2. Preparation and Characteristics of Nanocellulose Aerogels

Aerogel, as one of the highly porous scaffolds, is made from various kinds of organic or inorganic biomaterials and exhibits a large surface-to-volume ratio and highly interconnected pore structure [37]. Along with their great progress in the past decades, aerogels have been attractive for their utilization in biomedical applications, such as tissue engineering, drug delivery systems, and tissue regeneration. However, the high requirements on the tissue-engineered scaffolds accelerated the design and fabrication of bioactive aerogels by promoting the attachment, proliferation, and osteogenic differentiation of seeding cells, which played vital roles in their applications for BTE.

### 2.1. Isolation of Nanocellulose

Cellulose, as an insoluble and hydrophilic polysaccharide polymer containing a glucopyranose ring unit bound by a β-(1,4) glycosidic linkage [38], has a wide range of sources and can be extracted from plants, bacteria, and algae in nature [39,40]. The molecular formula of cellulose is (C6H10O5) n, consisting of β-D-glucose units. Each unit contains three hydroxyl groups, with one primary hydroxyl group located at C6 and two secondary hydroxyl groups situated at C2 and C3. The reactivity of these hydroxyl sites enables the modification and functionalization of cellulose. The degree of polymerization, n, varies depending on the source of cellulose as well as the methods employed for isolation and purification [41]. Nanocellulose is the nanoscale cellulose obtained by the physical and chemical treatment of cellulose. The preparation of nanocellulose materials from raw cellulose is divided into two stages. The first step is the purification that removes lignin, hemicellulose, and impurities from the raw material and the second step is to separate these pretreated cellulose materials to form a nanoscale cellulose material [42]. Hydrolysis plays a crucial role in pretreating cellulose by catalyzing the breakdown of β-D-glucose units into monosaccharides or oligosaccharides through water addition, with assistance from strong acids, alkalis, or enzymes [43]. Strong acids such as hydrochloric and sulfuric acid promote the acidic hydrolysis of cellulose to yield glucose and oligosaccharides. Under basic conditions, sodium hydroxide promotes alkaline breakdown [44].

### 2.2. Purification of Nanocellulose

Purification methods of cellulose include enzymatic hydrolysis, mechanical and chemical treatments including alkaline–acid pretreatment, oxidation, and ionic liquid treatment [45,46]. Cellulose can be oxygenated and separated at low temperatures using a CuCl_2_-NaClO_2_-MgCl_2_ solution, or through strong acid-based methods such as nitric acid and sodium hydroxide. These chemical oxidation methods can induce chemical reactions, such as carboxylation, hydroxylation, esterification, etc., thus altering the properties of cellulose and making it more convenient for subsequent processing and utilization [47]. The choice of purification method depends on the sources of cellulose. For example, the alkaline–acid pretreatment is suitable for soy hull fibers or wheat straw cellulose. The ionic liquid treatment can be selected when bagasse is used as a source of cellulose [48]. The method of enzymatic hydrolysis can separate fibers through the laccase degradation of lignin and hemicellulose. Mechanical treatment, including high-pressure homogenization, ultrasonic, freezing crushing, micro-jet mean and grinding methods, would generate a critical pressure at the center of the fibrous material and break the interaction between cellulose fibers [49]. Among them, the ultrasonic and high-pressure homogenization techniques have no significant effect on the physical and chemical properties of cellulose materials [50]. Although mechanical treatment has the above-mentioned advantages in the fabrication of nanocellulose materials, it is still powerless in scale-up processing. Consequently, many large-scale preparation methods of nanocellulose were developed, such as electrostatic spinning, melt-blown spinning, wet spinning, dry spinning, and gas foaming [25,51]. Gas foaming refers to a technique whereby an inert gas foaming agent is incorporated into the polymer phase, leading to the formation of gas bubbles inside 2D scaffolds via a series of chemical reactions. This results in the expansion of interconnected pores within the scaffolds [52]. The aerogels could also be prepared by the technology of gas foaming to reassemble the tightly packed 2D electrospinning nanofibers into fluffy 3D scaffolds with high porosity and large pores [35]. Although the 3D aerogels prepared by the gas foaming method showed great promise in BTE, there are still very few studies to fabricate nanocellulose-based aerogels via the technology of gas foaming. The subsequent research should focus on this field.

### 2.3. Various Kinds of Nanocellulose

Nanocellulose refers to nanoscale cellulose, and they comprise cellulose nanofiber (CNF), cellulose nanocrystalline (CNC), and bacterial cellulose (BC) [49]. CNF, with crystalline and amorphous regions, is obtained by the chemical removal of hemicellulose and then acid hydrolysis or mechanical treatment. The amorphous region of CNF contributes to its flexibility and plasticity, while the crystalline region contributes to its stiffness and elasticity [45]. CNC is a highly crystalline and needle-like structure obtained by sulfuric acid hydrolysis [48]. BC is produced by Gram-negative acetic acid bacteria from low molecular weight carbon sources which is completely free of lignin and hemicellulose [53].

The mechanical properties of CNF are higher than those of CNC and BC. Cellulose nanofibers with a higher mechanical strength can be obtained by sequential self-assembly strategies [54,55]. In addition, the self-assembly of nanocellulose might lead to inhomogeneous dispersion in non-polar solvents. The hydrogen bonds between the nanocellulose network are dynamic and could be broken and reorganized when they were stretched, which improved the performance of the nanocellulose materials [56]. As to the aerogels with a high CNF content, the porous structures might result in a higher density due to the continuous self-assembly.

### 2.4. Fabrication Steps for Nanocellulose Aerogels

**Figure 1:**
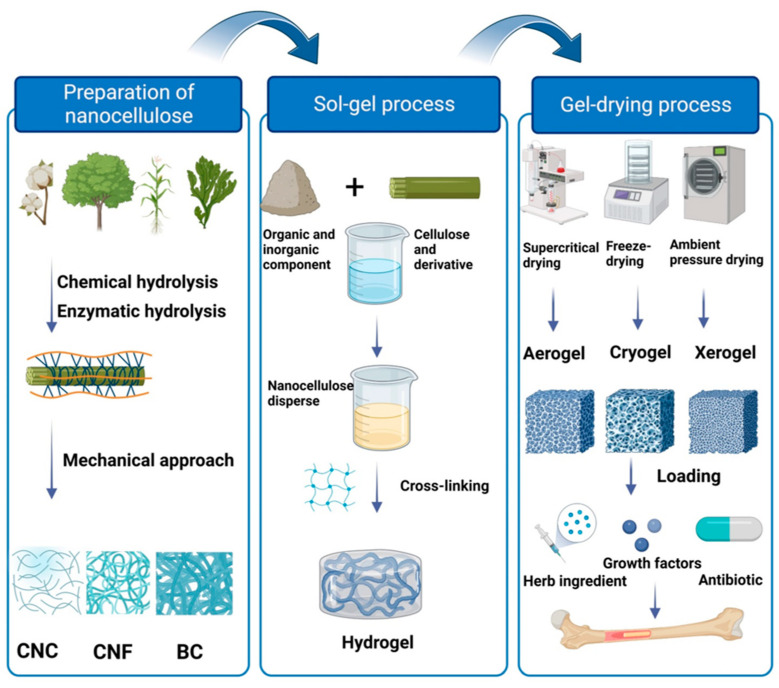
The scheme of fabrication of cellulose-based aerogels by sol–gel method. The first step is preparation of nanocellulose including purification by chemical and enzymatic hydrolysis and separation of these pretreated cellulose materials to form a nanoscale cellulose material by mechanical approaches. The second step is a sol–gel process including polymer dispersing in solvent and crosslinking stage and the third step is gel-drying process. Created with BioRender.com.

The preparation of nanocellulose aerogels generally includes three processes: preparation of a polymer in the solvent, sol–gel polymerization by crosslinking, and gel-drying [26,57,58] (Figure 1).

#### 2.4.1. Preparation of Nanocellulose Polymer Dispersion

The first step to constructing the nanocellulose-based aerogels is to prepare an aqueous nanocellulose-based suspension/gel by dispersing the raw nanocellulose materials into the dispersion. Due to the existence of an abundant active hydroxyl group on the surface of nanocellulose, it is easy to form intramolecular and intermolecular hydrogen bonds among the nanocellulose-based scaffolds [59]. Therefore, nanocellulose is difficult to disperse in an aqueous solution uniformly and tends to self-assemble and produce an entangled or 3D networks scaffold in the first step. To conquer this problem, some negatively charged groups can be introduced onto the nanocellulose surface to form a uniform nanocellulose dispersion in water by electrostatic repulsion [60,61]. For instance, a methylcellulose additive effectively eliminated delamination during the process of homogenization and prevented the aggregation of nanocrystals in the cellulose dispersion [62].

#### 2.4.2. Sol–Gel Transition

During the process of sol–gel transition, the stable nanocellulose was formed by transformation from a liquid polymer to a solid gel after chemical, physical, or enzymatic crosslinking. Crosslinking is a stabilization process which connects the functional groups of a polymer chain to another functional group through covalent bonds or non-covalent bonds, and finally leads to a network structure [63]. Crosslinking of the nanocellulose polymer can not only form a more stable 3D structure and improve the properties of the biomaterials in BTE, but also result in undesirable changes and cytotoxicity [64,65,66]. Chemical crosslinking through chemical reactions, producing irreversible covalent linkages between the cellulose molecules such as esterification reactions and salinization [67]. Physical crosslinking of nanocellulose began with the physical interactions between polymers such as ion–ion interaction, metal coordination, hydrogen bonding, host–guest interaction, p–p stacking, dehydration heat treatment, and ultraviolet treatment [67,68]. For example, inorganic salts and metal ions can be physically added to nanocellulose dispersions to form reversible crosslinks [69,70]. Compared with chemical crosslinkers, physical crosslinkers have a lower toxicity and higher biocompatibility. However, chemical crosslinkers could improve the mechanical properties of cellulose and are suitable for large bone defects [71]. An enzymatic crosslinking method utilizes specific enzymes to catalyze the polymer under specific conditions, and this process can usually be controlled by changing the temperature, pH, or ionic strength [72]. However, enzyme crosslinking cannot be scale-up used and is limited in the treatment of large bone defects due to its high cost (Table 1). If more than one type of crosslinking method was applied in fabricating cellulose aerogel, a more stable network structure could usually be achieved. This technology is called dual or multicrosslinking.

#### 2.4.3. Gel-Drying

Gel-drying refers to the replacement of aqueous in the nanocellulose gel by air to obtain an aerogel after the dispersion and gelation of nanocellulose. The key to successfully preparing nanocellulose-based aerogels is to maintain the porous structure during solvent removal. Different drying techniques, such as CO_2_ supercritical drying (scCO_2_), ambient pressure-drying [76], freeze-drying [74], phase separation [59,77], and gas foaming [78,79,80], have been used and produced different types of aerogels [26]. Among them, CO_2_ supercritical drying (scCO_2_) is the most versatile method for preparing aerogels. CO_2_ supercritical drying controls the temperature and pressure appropriately to make the solvent reach its critical point and for it to be converted from a liquid phase to gas phase. Since the solvent has no obvious surface tension in this process, it can produce aerogel materials with a more uniform structure while maintaining its 3D network skeleton structure [81]. The disadvantages of supercritical drying are the requirement for expensive high-pressure equipment and extremely harsh processing conditions.

Freeze-drying is essentially a sublimation drying process, which can reduce gel shrinkage when compared with ambient pressure-drying and supercritical drying [76,82]. As the two-phase contact between gas and liquid needs to be avoided in this process, it can effectively prevent the capillary pressure during the drying process to produce highly aligned and controlled porous aerogels. Compared with CO_2_ supercritical drying, cellulose aerogel processed by freeze-drying has a higher compression modulus and higher yield behavior at the same density [83]. However, scaffolds fabricated by freeze-drying exhibited lamellar and honeycomb structures rather than real nonporous structures, due to the freeze–thaw and solvent exchange processes reducing the aggregation and hydrogen bonding between CNF [84,85]. Due to its distinct advantage of sustainable and low cost, freeze-drying is still the most utilized gel-drying technology in the fabrication of nanocellulose aerogels [26,59,76]. Different from the method of freeze-drying, phase separation required no solvent exchange and could effectively reduce the gel-drying time of aerogels [77,85]. On the other hand, aerogels prepared by ambient pressure-drying showed a smaller pore size, large shrinkage after gelation, and fragile structure.

## 3. Application of Nanocellulose-Based Aerogels in BTE

Bone is a highly mineralized, vascularized, and connective tissue that has remarkable mechanical strength, which provides fracture toughness and load-bearing ability to protect internal organs [86]. The ideal bone substitutes should mimic the microstructure of native bone tissues and offer a biological environment for bone regeneration and tissue repair. Moreover, the design and preparation of hybrid nanocellulose aerogels should fully understand the structure and compositions of natural bone tissue.

### 3.1. Microstructure of Bone ECM

The main inorganic component of bone is hydroxyapatite (HA) crystals, which are embedded in the extracellular matrix (ECM) of bone. As the organic component of bone tissue, bone ECM is mainly composed of type I collagen fibers and serves as an inductive template for bone repair [87,88]. The mineral hydroxyapatite crystals deposit along the long axis of collagen type I fibers and present a hierarchical deposition within zones between collagen fibrils at the nanoscale [89,90,91].

**Figure 2:**
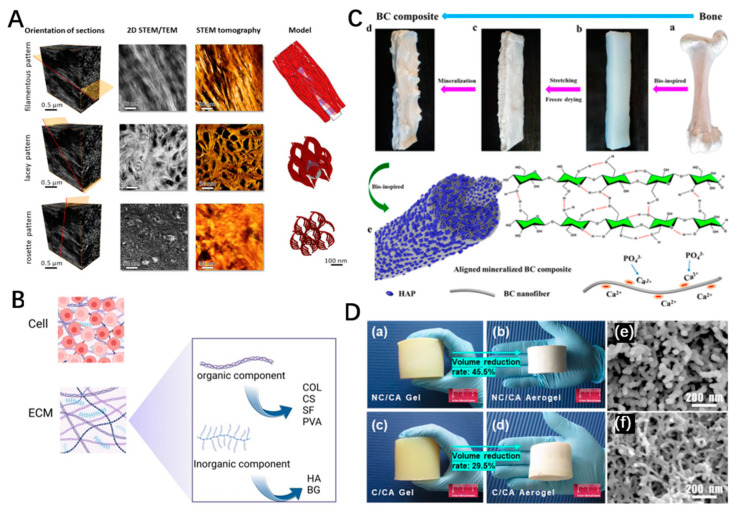
(**A**) Patterns of the bone mineral organization in different directions [89]. Copyright 2018, Science. (**B**) Composite preparation of bone biomimetic material. Created with BioRender.com. (**C**) Schematic illustration of the mineralization process [102]. Copyright 2019, American Chemical Society. (**D**) (**a**,**b**) Pure chitosan gel and aerogel. (**c**,**d**) CNC-modified chitosan gel and aerogel. (**e**,**f**) Microstructure of pure chitosan aerogel and CNC-modified chitosan aerogel [103]. Copyright 2021, American Chemical Society.

The minerals of bone tissue are hierarchically assembled from nanoscale [89]. Before mineralization, the organic phase of bone has been assembled, which can finely regulate crystal nucleation and growth. Needle-like mineral particles coalesce horizontally into platelets, neither inside nor outside the fibers, but form fractal-like hierarchical bone architecture with continuous intersecting fibers [89]. The mineralized collagen fibers on the microscopic scale are arranged in a complex hierarchical structure. At the macro level, most bones contain helical patterns in their anatomical shapes to increase adaptation to force. At the micro level, the spiral secondary bone itself is formed by concentric slices of mineralized collagen fibers. In terms of scaffold designing, biomimetic approaches, which can simulate molecular structural and biocompatibility with complex natural bone tissue [92,93,94,95] (Figure 2A), have gained increasing attention. By exploiting the unique properties of the pure or composite nanocellulose scaffolds, it is possible to improve the properties of the biomimetic materials with controlled and layered structures in nanostructures [55].

Electrospinning offers clear advantages for the preparation of scaffolds based on nanocellulose, including control over composition, structural design, and functional expansion [96,97]. It is a promising method for producing 3D aerogels in BTE and for mimicking the extracellular matrix (ECM) [35,98,99,100]. The core–shell structure of electrospinning is composed of PHB/G and PHB/G/Fe_3_O_4_ compositions, which result in lower melting points compared to pure PHB scaffolds. The resulting hybrid scaffolds have a lower crystallinity and are non-toxic, with the added benefit of high saturation magnetization in the magnetite composite scaffolds, which makes them well suited for biomedical applications [101]. In addition, gas foaming is a process that involves introducing inert gas foaming agents into the polymer phase, generating gas bubbles inside the 2D scaffolds via subsequent chemical reactions to expand the interconnected pores within the scaffolds [50]. Aerogels can also be prepared using gas foaming technology, which involves reassembling tightly packed 2D electrospinning nanofibers into fluffy 3D scaffolds with high porosity and large pores [35]. While 3D aerogels produced by gas foaming show great promise in BTE applications, there have been very few studies on fabricating nanocellulose-based aerogels using this technology. Therefore, future research should focus on this area.

### 3.2. Nanocellulose Aerogel Alone

Since 1971, when the first generation of a cellulose-based aerogel with a large specific surface area was fabricated, various studies have been performed to evaluate the toxicity, antibacterial properties, and mechanical properties of nanocellulose aerogels and provide a theoretical basis for their application in BTE [83,104,105,106]. Li et al. prepared a CNC-based aerogel by direct ink writing and freeze-drying and proved that the resulting aerogel exhibited dual porous and controllable structures [107]. The main disadvantage of the CNC-based aerogels is obviously their brittleness, which would lead to structural damage during cell incorporation and growth. Optimizing the crosslinking method might improve mechanical performance by adding 2.5 wt% polyamide-epichlorohydrin (kymene) into the nanocellulose polymer dispersion before crosslinking. This increased the Young’s modulus of the composite aerogel from 7 MPa to 8.94 MPa [107]. Epoxypropane exhibits significant cytotoxicity, and its linear structure with bulky side chains limits its degradation compared to other types of chemical crosslinking agents, which restricts its usage in tissue engineering [63,108]. In another study, Osorio et al. grafted a hydrazide group onto a carboxylic acid group to form a hydrazone linkage on the surface of a CNC-based aerogel and proved that the prepared cellulose aerogel presented an excellent flexibility, high porosity, and osteoconductive properties after chemical crosslinking [64].

### 3.3. Nanocellulose-Based Composite Aerogels

Due to the existence of hydrogen bonds, nanocellulose can not only be self-assembled itself, but also assembled with other polymer materials. The aerogels with cellulose alone presented the disadvantages of hydrophilicity and poor osteoconduction [109,110]. In order to overcome these drawbacks and preserve their inherent superiorities, the composite fabrication methods of nanocellulose aerogels have received more and more attention, as the mechanical properties, biodegradability, bioactivity, and superior biological properties of bone scaffolds are adjusted by combining cellulose with different organic and inorganic compounds (Figure 2B) [111,112]. The contents regarding the combination of nanocellulose with other materials are summarized as follows (Table 2).

#### 3.3.1. HA–Nanocellulose Aerogel

Scaffolds with both organic and inorganic components could biomimic the microstructures of natural bone, which not only facilitates the proliferation of osteoblast lineage cells but also provides an optimal microenvironment for the formation of blood vessels. Traditionally, biomimicking the inorganic phase of bone tissues has mainly focused on inorganic materials such as nano-silicate particles, calcium phosphate, and bioactive glasses.

As one kind of environment-friendly biomaterial, hydroxyapatite (HA), with an excellent biocompatibility, constitutes the inorganic phase of bone and could release various osteoconductive ions into the surrounding environment [95,122,123]. However, the shortcomings of HA, such as low absorption rate in vivo, low crack resistance, and poor bone stimulation, limit its clinical application [124]. Adding HA to cellulose-based aerogels can enhance the mechanical properties to construct organic–inorganic aerogels. Huang et al. attached an in situ HA coating at approximately 10 nm onto the surface of CNC and then crosslinked it with polymethyl vinyl ether-malonic acid (PMVEMA) and polyethylene glycol (PEG) to enhance the mechanical properties of the composites. Fourier-transform infrared spectroscopy (FTIR) and nuclear magnetic resonance (NMR) showed esterification reactions occurring between CNCs, HAP, PMVEMA, and PEG. The results showed that the attachment of HA increased the compressive strength of the resulting scaffold up to 41.8 MPa, which provides a broad potential in the development of BTE scaffolds [113]. Cheng et al. have also fabricated an HA–BC aerogel and performed the in situ mineralization by embedding the aerogel into the CaCl_2_ and K_2_HPO_4_ solutions, and the results demonstrated that the composite aerogel with excellent biocompatibility enhanced the mechanical properties and biomimicked the structure of natural bone (Figure 2C) [102]. The above-mentioned studies proved that the incorporation of HA into the nanocellulose aerogels could not only enhance their mechanical properties but also functioned as a promising template for biomimetic mineralization. Moreover, advanced preparation techniques such as 3D printing could be used to orderly deposit HA layers onto the collagen fibers to mimic the microstructure of the native bone tissue due to the urgent requirement of biomimic theory.

#### 3.3.2. Bioactive Glasses–Nanocellulose Aerogel

Bioactive glasses (BG) can release calcium and phosphate into the surrounding environment and result in the deposition of HA on the surface of biomaterials after the transplantation of bioactive glass in vivo. Bioactive glasses-based bone substitutes have been widely applied in BTE. Kamel et al. prepared a nano-fibrillated cellulose aerogel loaded with strontium borate-based bioactive ceramic particles and rosuvastatin to treat the extraction socket [34]. The results showed that the composite aerogel exhibited excellent mechanical properties and promoted the proliferation of MG-63 cells, which exhibit a promising material for the preservation of dental sockets.

#### 3.3.3. Collagen–Nanocellulose Aerogel

As we all know, the organic phase of native bone tissue functions as the hierarchical skeleton and plays a crucial role in biomineralization. Commonly used biopolymers include chitosan (CS), collagen, cellulose, etc. All have been proven to be suitable platforms that mimic inorganic phases of bone and substitutes to fabricate composite scaffolds with a similar structure and composition to native bone [125]. As the main component of the organic phase of bone tissue, collagen (Col) in native bone could be treated as a template for biomineralization that controls the orientation and shape of HA crystal by providing nucleation sites. Then, the in vivo biomineralization process happens and leads to the nucleation and growth of HA nanocrystals along the axial of the Col fibers [126]. In recent years, many studies have focused on the preparation of cellulose biomimetic scaffolds. For instance, a biomimetic collagen–carboxymethyl cellulose/hydroxyapatite scaffold was prepared by He et al. through a biomolecular template of collagen–carboxymethyl cellulose, and the scaffold presented good biocompatibility. By controlling the ratio of collagen to carboxymethyl cellulose in the template, the osteoinductivity, the osteoconductive, and the mechanical strength of composites could be changed and adjusted according to the requirement of BTE [127]. Xu et al. prepared nanocellulose–collagen (COL)-nanohydroxyapatite(n-HA) organic–inorganic hybrid aerogels by adding collagen and HA into cellulose aerogels and found that the composite aerogels exhibited a porous 3D structure with high compressive strength, excellent osteogenesis, and angiogenesis abilities both in vitro and in vivo [119]. Based on the above-mentioned research, it is safely concluded that the organic–inorganic hybrid materials based on the combination of Col and nanocellulose could construct multilevel bionic scaffolds from macro to micro and present great potential for repairing bone defects.

#### 3.3.4. Chitosan–Nanocellulose Aerogel

Chitosan (CS), with a structural similarity with glycosaminoglycan, has excellent osteoconduction ability [128,129]. In a study, high-pressure homogenization and freeze-drying technologies were utilized to fabricate CNF-based and chitosan-based composite aerogels. The results showed the CNF aerogels exhibited the highest porosity, lowest density, and worst mechanical properties. However, adding chitosan into CNF can not only significantly improve the mechanical properties but also reduce the water absorption of the composite aerogels [117]. In another study, Matinfar et al. prepared biphasic and triphasic calcium phosphate fiber-reinforced CS- carboxymethyl cellulose (CMC) porous scaffolds by a freeze-drying method [114]. The broad band observed in the chitosan spectrum between 3367–3449 cm^−1^ corresponds to the stretching vibration of N–H and O–H groups. In addition, the CMC powder spectrum exhibited distinctive bands at 1602 cm^−1^, 1424 cm^−1^, and 1330 cm^−1^, which are characteristic of carboxyl, methyl, and hydroxyl groups, respectively. Furthermore, a band at 1057 cm^−1^, attributed to the stretching vibrations of -CH2OH, was also observed. The biphasic fiber was composed of HA and triclinic apatite, and the triphasic fiber was composed of HA, β-tricalcium phosphate, and calcium pyrophosphate. After adding CMC to CS aerogel, its mechanical properties and cell viability were significantly improved. After adding CS into CMC aerogel, the viability of cells attached to the composite aerogels was significantly improved (Figure 2D) [103]. However, its mechanical properties need to be further improved. Thus, the incorporation of the organic phase into the CS–CMC aerogels further enhanced their mechanical properties and effectively solved the above-mentioned problem.

#### 3.3.5. PVA–Nanocellulose Aerogel

Polyvinyl alcohol (PVA) is also a favorable biopolymer. With insufficient mechanical strength, which is significantly lower than natural bone, PVA alone is not suitable to be fabricated into BTE substitutes. Incorporation of PVA into the nanocrystalline cellulose scaffolds could also solve this problem and tailor their biological performance. Zhou et al. synthesized a PVA/CNFs/gelatin hybrid aerogel by the utilization of gelatin as the crosslinking agent. The modulus of the PVA/CNFs/gelatin aerogels is 1.65 MPa, significantly higher than those of the pure CNF and PVA/CNF aerogels [109]. Cataldi et al. combined nanocrystalline cellulose with PVA to fabricate a composite scaffold with enhanced tensile stress, contributed by the involvement of the nanocrystalline cellulose. However, the incorporation of an excessive amount of nanocrystalline cellulose also led to the agglomeration of nanoparticles and decreased the tensile stress of the composite scaffold [130]. Liu et al. prepared CNFs/PVA/montmorillonite aerogels and investigated the effects of crosslinkers (borax and glutaraldehyde) on the formation of the interface bonding and porous network. The results proved that glutaraldehyde crosslinking resulted in larger and looser pores of the composite aerogels as compared with those prepared by the borax crosslinking method [131]. Therefore, adding nanocellulose would increase the mechanical performance of the composite scaffolds, whereas incorporation of PVA enhances their biocompatibility.

#### 3.3.6. SF–Nanocellulose Aerogel

Silk fibroin (SF), with a favorable biocompatibility and noncarcinogenic ability, is extracted from silkworm cocoons and has the ability to promote preosteoblasts proliferation and MSCs osteogenic differentiation, demonstrating favorable advantages in bone regeneration [132,133]. However, its short absorption times and low mechanical properties limited the application of SF in the BTE field due to the high requirements for bone substitutes and the relatively long healing process of bone tissues. After the combination of SF and nanocellulose materials with relatively longer absorption periods and higher mechanical properties than SF, SF/nanocellulose composites exhibit the advantages of both SF (good biocompatibility, easy degradation, and excellent osteoinductive ability) and nanocellulose (remarkable mechanical strengths and long absorption time), making them great prospects for functional applications in BTE. Chen et al. prepared mineralized self-assembled silk fibroin (SF) –cellulose composite aerogels with an interpenetrating network by freeze-drying. In situ mineralization was then performed to control the nucleation and growth of n–HA crystals onto the surface of the composite aerogels [116]. After the mineralization of HA, the zeta potentials of the cellulose aerogel and SF/nanocellulose composite decreased from −11.1 mV and −26.3 mV to −6.3 mV and −4.1 mV, respectively. These zeta potentials are close to the −5.8 mV of n–HA. The results show that mineralized SF–cellulose composite aerogels have a good microstructure such as ideal cancellous bone, moderately adjusted compressive strength, and high degradative rate in vitro. In addition, it can also promote the proliferation of human embryonic kidney cells (HEK293T) which has potential in BTE [116]. Although only a few studies have focused on SF–cellulose-based aerogels and their application in BTE fields, there is still an attractive potential for nanocellulose-based aerogels in repairing bone defects.

## 4. Nanocellulose Aerogels-Based Controlled Releasing System for BTE

Cellulose-based aerogels can also serve as antibiotics, bioactive factors, and herbal ingredient carriers to affect the adhesion, proliferation, and migration of seed cells and enhance osteogenesis and angiogenesis for bone regeneration [134,135]. According to the literature, 22% of aerogels have been utilized for drug loading, while only 19% of aerogels have been used for tissue engineering in biomedical applications [112]. The aim of the nanocellulose aerogel-based controlled-release approaches is to maintain the stability of the cargo and the concentration within the therapeutic window for an extended period, further increasing therapeutic effects by lowering the loading dosage and reducing side effects. Due to a large surface area, the nanocellulose aerogel-based controlled releasing systems could effectively absorb the loading cargo and release them into the surrounding environment [136]. After combing the nanocellulose aerogels and drug/bioactive molecules, the nanocellulose aerogel-based control releasing system not only had intrinsic properties of the nanocellulose-based aerogels such as low density, high space surface area, and high mechanical strength, but also benefited from the features of drug/bioactive molecules such as osteogenic and osteoinductive abilities [21].

### 4.1. Antibiotic, Growth Factors, and Chinese Herbal Medicine Delivery

**Figure 3:**
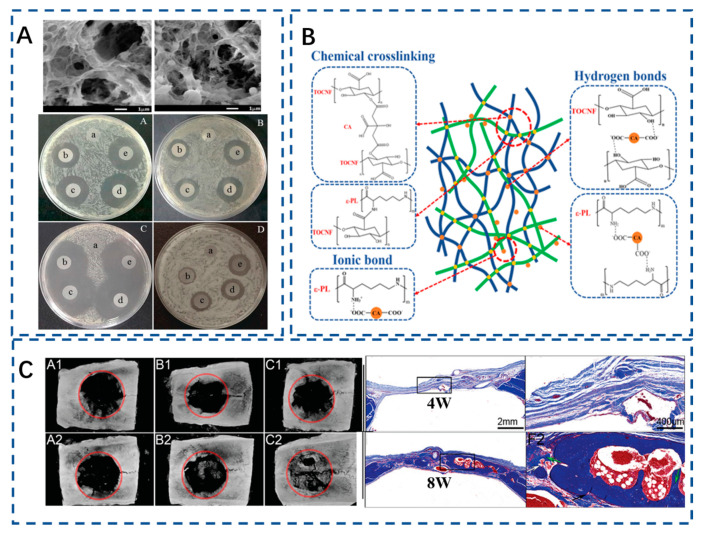
(**A**) SEM images of amoxicillin loaded into cellulose aerogels and optical images of inhibition zones of cellulose aerogels [140]. Copyright 2018, Molecular Diversity Preservation International. (**B**) Characterization of antibacterial aerogel based on ε-poly-L-lysine/nanocellulose by using citric acid as crosslinker [120]. Copyright 2022 Elsevier. (**C**) Representative radiographs of cranial bone defects. (**A1**/**A2**), without treatment (4 w/8 w); (**B1**/**B2**), 3D hybrid nanofiber aerogel (4 w/8 w); (**C1**/**C2**), E7-BMP-2 peptide loaded 3D hybrid nanofiber (4 w/8 w); and d Masson’s trichrome stained images of unfilled defect (4 w) and E7-BMP-2 peptide loaded 3D hybrid nanofiber aerogel (8 w) [147]. Copyright 2018, Wiley.

Various kinds of antibiotic are typical delivery cargos and have been widely applied in biomedical research in recent years [137,138,139]. For example, a CNF aerogel-based controlled releasing system loaded with amoxicillin had been prepared by Ye et al. and showed a controlled releasing performance of amoxicillin. The results of an in vitro antibacterial experiment demonstrated that the prepared controlled releasing system exhibited an antibacterial activity and the antibacterial effects increased with the content of the loading amoxicillin (Figure 3A) [140]. Wang et al. also designed an antibacterial aerogel based on a TOCNF/∂-poly-l-lysine (∂-PL) crosslinked network, and the results showed that the fabricated controlled releasing system exhibited a good degradability and excellent antibacterial efficiency (up to 99.9%), thus confirming its potential in BTE (Figure 3B). Nevertheless, the grafting and esterification reactions have slightly reduced the thermal stability of the cellulose aerogel. This is potentially caused by the disruption of the hydrogen-bond network between ε-PL and cellulose upon its incorporation [120].

Various kinds of cells and growth factors were also loaded into the nanocellulose aerogels to treat bone defects to achieve rapid tissue repair. The potential growth factors which could be control released by the nanocellulose aerogels for BTE include bone morphogenetic protein (BMP), vascular endothelial growth factor (VEGF), stromal cell-derived factor-1 (SDF-1), sclerostin monoclonal antibody, etc. [119]. Among them, BMP and VEGF are commonly utilized factors to promote osteogenesis and angiogenesis. Recent studies have shown that BMP plays a crucial role in the initial stage of osteogenesis and can still play a role in promoting osteogenesis 16 days after complete release [141]. BMP-2 and BMP-7 have shown satisfactory osteoinduction and osteoconduction in clinical studies and relevant products containing rhBMP-2 and rhBMP-7 have been approved by the Food and Drug Administration (FDA) [142]. However, BMP-2 has some dose-related side effects including ectopic one formation, osteoclast-mediated bone resorption, inappropriate adipogenesis, and unwanted immunogenic responses of the host [143,144]. Notably, side effects of BMP-2 were expected to be reduced by optimizing the total amount of the loaded factors and controlled releasing approaches [145]. In addition, short chain BMP-2 peptide mimics the activity of BMP-2 protein by binding to cell receptors. In addition, it has been reported that calcium-conjugated BMP-2 peptide with a controlled release rate can significantly improve the binding ability of BMP-2 to the surface of hydroxyapatite (HA) to enhance the efficiency of bone mineralization and reduce side effects (Figure 3C) [146,147]. Moreover, a study investigated BC scaffolds loaded with a low dose of BMP-2 and primary mouse mesenchymal stem cells (C3H10T1/2 cells). The results showed that BMP-2 induced the adhesion and proliferation of cells [17]. VEGFs play a critical role in angiogenesis; QK peptide is a VEGF mimetic peptide that promotes angiogenesis and bone regeneration by activating VEGF receptors [148]. John et al. prepared a poly(𝜖-caprolactone)/gelatin/gelatin methacryloyl nanofiber aerogel coupled with QK peptide to regulate the formation of microvascular networks in seed endothelial cells [149]. In conclusion, the above-mentioned nanofiber aerogel can be applied in combination with signal molecules and cells to provide an ideal microenvironment for cell infiltration and proliferation for tissue repair.

The bioactive molecules of traditional Chinese medicine such as icariin, drynaria, salvia miltiorrhiza, etc., have a certain role in promoting bone repair, and the sustained release of these traditional Chinese medicines by the nanocellulose-based aerogels might have promising potential for bone regeneration [150,151]. Resveratrol (Res) is a phenolic compound with a chemical formula of C14H12O3. It has anti-inflammatory and antioxidant effects, but is known for its limited solubility in water, which can make oral administration challenging [152]. Qin et al. prepared resveratrol-loaded TEMPO-oxidized cellulose aerogels by a freeze-drying method. FTIR spectroscopy revealed a wide peak at 3192 cm^−1^, indicating a Res–OH stretching vibration and the presence of intramolecular hydrogen bonding. Furthermore, a significant alteration in the hydroxyl stretching band has been observed with an increase in the cellulose content, suggesting the existence of intermolecular hydrogen bonding between cellulose and Res’s hydroxyl groups [153]. The study proved that the resveratrol-loaded TEMPO-oxidized cellulose aerogels showed excellent stability in PBS and the simulated gastric fluid and could stably release resveratrol, indicating that it has good potential in the treatment of osteoarthritis [153].Puerarin is a natural isoflavone compound commonly extracted from kudzu roots, which has anti-inflammatory and antioxidant effects in tissue engineering by binding with endotoxin and synergistically destroying the bacterial membrane structure [154]. FTIR and XRD analyses showed that the hydroxyl groups of puerarin were bound to the amino groups of chitosan. Moreover, puerarin has also been shown to promote new bone formation in β-tricalcium phosphate osteoblast complexes in vivo [155]. However, there is no research to study puerarin-loaded cellulose aerogels. Therefore, the relevant research remains to be further studied.

### 4.2. Smart Drug Delivery System

**Figure 4:**
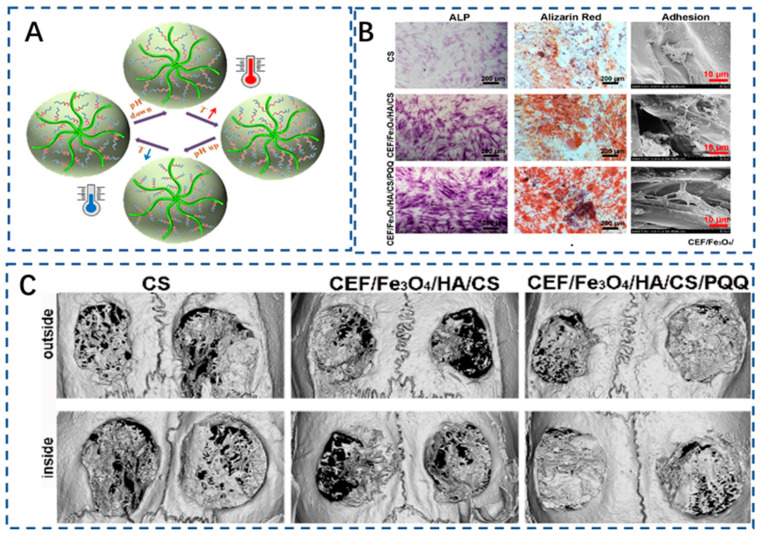
(**A**) CNF-PEI-NIPAM exhibits a dual-responsive mechanism to temperature and pH changes. [157] Copyright 2020 Elsevier. (**B**) ALP activity and Alizarin Red staining showing that magnetic-oriented CS–cellulose promoted BMSC osteogenic differentiation and adhesion [161]. Copyright 2022 Elsevier. (**C**) Micro-CT demonstrated that magnetic-oriented CS–cellulose significantly facilitated the healing of cranial bone defect, [161]. Copyright 2022 Elsevier.

The smart responsive nanofibrous cellulose aerogel has broad application prospects and development potential in tissue engineering. It can respond intelligently to external factors such as temperature, pH value, light, and magnetism, thus enabling precise control of the release of active substances and cell growth behavior in the aerogel [29,156]. It can facilitate tissue growth and wound healing, providing better conditions for tissue repair and regeneration. Liang and her colleagues devised and produced cellulose nanofibers that are biocompatible and smartly dual-responsive by attaching a temperature and pH-responsive polymer (PEI-NIPAM: polyethyleneimine-N-isopropylacrylamide) onto CNF-COOH. CNF-PEI-NIPAM aerogels, and demonstrated excellent dual-responsiveness to pH and temperature, with over 99% antibacterial activity against E. coli. Additionally, under conditions of 37 °C and pH 3, the drug-loading capacity of the CNF-PEI-NIPAM aerogel for doxorubicin reached 330.12 mg/g, with an accumulated release rate of 59.45%. The wettability of CNF-PEI-NIPAM is mutually promoted under low-temperature and acidic conditions, while it is mutually inhibited under high-temperature and alkaline conditions. Its hydrophilicity increases with temperature, while its wettability decreases with increasing pH (Figure 4A). When the pH value increases from 1 to 9, CNF-PEI-NIPAM changes from hydrophilic (contact angle, CA = 49.1°) to hydrophobic (CA = 135.5°) due to its large number of pH responsive amino groups [157]. This novel temperature and pH dual-responsive smart nanofiber cellulose provides a new avenue for the design of novel bone tissue repair materials.

The use of magnetic nanocellulose in the biomedical field has promising applications, including drug delivery, hyperthermia, and tissue engineering. Recent studies have shown that magnetic nanocellulose can significantly improve drug delivery efficiency, leading to greater therapeutic benefits with lower dosages. With the addition of drugs and magnetic responsiveness, nanocellulose can achieve precise and rapid drug delivery control [158,159]. This can improve drug bioavailability and efficacy while reducing side effects and waste. Iron oxide nanoparticles have been widely investigated and applied in the preparation of magnetic nanocellulose due to their superior biocompatibility and low toxicity. Arifa Naznin et al. prepared cellulose-loaded magnetic iron oxide nanoparticles. The swelling capacity of the particles increased from 155.0% to 159.5%. Furthermore, the drug loading and release time of metronidazole also increased [160]. According to research findings, it has been demonstrated that n–HA was deposited on the surface of CNF, and magnetic nanoparticles on the CNF were oriented on the surface of chitosan under the action of a magnetic field. The oriented cellulose fibers enhance the compressive properties of the scaffold, while the n–HA on the oriented cellulose fiber surface promotes the formation of new blood vessels and accelerates chondrogenesis, ultimately guiding bone growth orientation and promoting bone activity [161] (Figure 4B,C). Ultrasound has emerged as a promising tool in the field of tissue engineering due to its capacity to modulate cellular behavior, enhance cell proliferation and migration, and induce changes in extracellular matrix production. For instance, low-intensity pulsed ultrasound (LIPUS) has been demonstrated to stimulate MC3T3-E1 proliferation and differentiation [162].

### 4.3. Modification of Nanocellulose-Based Aerogels

Commonly, modification after aerogel fabrication can be conducted by surface coating and chemical vapor deposition [163,164,165]. Based on the limitations of the cellulose aerogels in BTE, this part mainly introduces hydrophobicity modification and mechanical property enhancement. These modification methods might be effective ways to adjust the surface chemical structure and broaden its application in BTE.

#### 4.3.1. Enhancement of Mechanical Properties

The mechanical properties of cellulose-based aerogels play a vital role in bone repair, and the selection of precursor materials, crosslinking methods, and modification after preparation all affect the mechanical properties of nanocellulose-based aerogels [166]. Drawing on the recent literature, it was discovered that there exists an inverse relationship between the porosity and mechanical strength of cellulose aerogels [167]. More specifically, as the porosity of cellulose aerogels increases, their mechanical strength tends to decrease, and vice versa. This phenomenon can be attributed to the fact that when the porosity is high, more voids are present within the material, which results in a decrease in the contact area between the cellulose fibers and an increase in the pore size, ultimately reducing the structural integrity of the material. In contrast, in the case of a lower porosity, the reduced number of voids facilitates a higher effective area of contact between fibers, thereby increasing the mechanical strength of the final product. Therefore, achieving a balance between the porosity and mechanical strength of cellulose aerogels is of utmost importance for their effective application in the field of tissue engineering. Crosslinking strategies can functionally modify the mechanical, biological, and degradable characteristics of nanocellulose aerogels with a specific composition and structure [63]. The mechanical properties of cellulose-based aerogels play a vital role in bone repair, and the selection of precursor materials, crosslinking methods, and modification after preparation all affect the mechanical properties of nanocellulose-based aerogels [168]. Krishnakumar et al. published a systematic review and summarized the limitations and future development potential of different types of biocrosslinking methods in BTE. The results showed that chemical crosslinking significantly enhanced the mechanical properties of cellulose aerogels when compared with those of physical crosslinking [169]. Traditional chemical crosslinkers such as glutaraldehyde (GA), genipin (GP), tannic acid (TA), citric acid (CA), and hexamethylenediamine (HMDA) could also improve the degree of gelation and their mechanical performance [71,73,74,170].

The commonly utilized surface modification methods of nanocellulose-based aerogels include esterification, salinization, surface coating, and graft copolymerization [171,172,173]. Esterification and etherification are the most common derivatization modifications of cellulose (Table 3). A modification technique using 2,2,6,6-Tetramethylpiperidine1-oxyl (TEMPO) oxidation was recently developed to defibrillate raw cellulose and create a modified aerogel with an improved mechanical performance [174]. TEMPO/NaBr/NaClO was added to the oxidation of natural cellulose in an aqueous solution at pH 10. Most of the C6-hydroxyl groups on the surface of crystalline cellulose microfibers were converted to C6-sodium carboxylate groups [175]. The TEMPO-oxidized wood cellulose fibers formed fully individualized TOCN dispersed in an aqueous solution after mechanical decomposition. The tensile strength and elastic modulus of the modified cellulose fibers increased to 200–300 MPa and 6–7 GPa, respectively [176]. Zheng et al. grafted polyethylene glycol (PEG) onto CNC to construct PVA–CNC composites and found that the composites exhibited a higher stress transfer efficiency and better stiffness strength when compared with the ungrafted composites [177]. After adding 1% CNC–PEG as the reinforcing filler, the ultimate strength and stiffness of the composites were increased by 40.4% and 34.2%, respectively [177].

#### 4.3.2. Enhancement of Their Osteogenic Ability

Enhancing the osteogenic induction activity of cellulose aerogels can be achieved by simulating the ECM of bone, enhancing vascularization, and surface modification.

**Figure 5:**
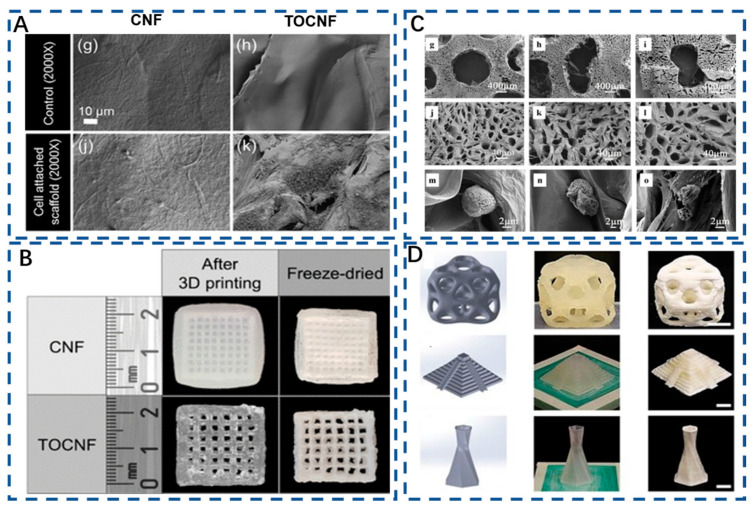
(**A**) Cardiomyocyte attachment and morphology on CNF (**g**,**j**) and TOCNF (**h**,**k**) [183]. Copyright 2019 American Chemical Society. (**B**) CNF, TOCNF scaffolds in the wet state soon after printing and after freeze-drying [183]. Copyright 2019 American Chemical Society. (**C**) (**g**–**l**) SEM images of CNF/PEGDA scaffolds with different Poisson’s ratios and (**m**–**o**) SEM images of cell adhesion after inoculation on CNF/PEGDA scaffolds [118]. Copyright 2021, Molecular Diversity Preservation International. (**D**) DIW 3D printed models. Displayed scale bars are 1 cm [107]. Copyright 2017, Nature.

Scaffolds of cellulose and its derivatives have been extensively studied in bone tissue regeneration due to their renewable and biodegradable properties. The multifunctionality of the nanocellulose-based aerogels are attributed to the involvement of new components into the nanocellulose-based aerogels system, which enhanced their osteogenic abilities. Recent studies have shown that carboxymethyl cellulose could induce osteogenic differentiation and it has great potential in the study of tissue engineering scaffolds [127]. A novel surface modification method was utilized to deposit cell-derived proteins onto the surface of BC and the results proved that this kind of surface modification increased the water contact angle and improved the mechanical strength, cell adhesion, and mitochondrial activities [180]. Osorio et al. also fabricated a hydrazone crosslinked CNC aerogel by adding aldehyde and hydrazide functional groups onto CNC, allowing for the formation of a hydrazone bond, and facilitation of bone regeneration [64]. Thus enhancing the osteogenic activity of cellulose aerogels,

Notably, the 3D printing of cellulose-based aerogels provides a hierarchically composite geometry with excellent controllability of personalized design and macroscopic structure [181]. The use of 3D printing can also enable organic–inorganic cellulose aerogels to perform hierarchical deposition, improve the mechanical strength of materials to suit irregular defects, and fabricate layered biomimetic scaffolds [66,182]. The use of 3D printing bone biomimetic preparation can improve the osteogenic ability of biomaterial. For example, a CNF and polyethylene glycol diacrylate (PEGDA) composite aerogel with adjustable Poisson’s ratio was prepared by 3D printing and provided dynamic stress environments for the differentiation of bone mesenchymal stem cells (BMSC) [115]. In another study, Tang et al. also prepared a PEGDA–CNF aerogel and evaluated its chondrogenic-inductive characteristics. The results showed that the scaffolds could facilitate the proliferation and chondrogenic induction of BMSC (Figure 5) [118]. All in all, 3D printing is expected to achieve a good balance of other functional characteristics while achieving rapid and large-scale production.

#### 4.3.3. Improvement in Hydrophilicity

Scaffolds for BTE require moderate hydrophilicity to promote tissue infiltration and cell attachment, but scaffolds with too much hydrophilicity are easy to collapse before the formation of bone tissues. Nanocellulose aerogels with extensive hydrogen bonds and hydroxyl on their surface would result in excessive hydrophilicity and cannot maintain their stability in vivo [45]. To enhance the stability of nanocellulose aerogels, the hydrophobicity of nanocellulose needs to be promoted by gel impregnation or surface coating [163]. Polydopamine (PDA) can be coated on the surface of various materials through self-polymerization under aqueous conditions to improve scaffolds’ hydrophobicity and mechanical property [177,184,185]. Tannic acid (TA), a low-cost natural polyphenol, can also be coated on nanomaterials to decrease the hydrophobicity of nanocellulose scaffolds through surface coating [56,186].

## 5. Challenges and Outlook

At present, although academic activity on nanocellulose-based aerogels has flourished in recent years, few have been transformed into clinical applications. The side effects of polymeric nanoparticles including nanocellulose on cells, which is not observable within a short time period, might account for this. In vitro experimental studies concerning the toxicity of nanocellulose-based aerogels need to be conducted for a long time to clarify their safety. Moreover, the optimal balance between biocompatibility, osteoinduction, and mechanical properties remains a major challenge for the application of nanocellulose-based aerogels in BTE. The development and application of nanocellulose-based aerogels can be accelerated by the incorporation of the promising organic polymer and enhancement of their osteogenic induction ability.

As to the drug releasing systems based on cellulose aerogels, the drug releasing time and rate have a close connection with the special properties, thermal, or pH response of carriers and need to be precisely controlled. Thus, it is urgent for researchers to develop drug-loaded cellulose aerogels with an intelligent response for BTE. The use of magnetic fields can enhance the targeting and delivery of therapeutic agents to specific sites, while minimizing their systemic toxicity. Furthermore, the integration of magnetic nanoparticles into cellulose matrices can lead to improved mechanical and physical properties, which can enhance their utility in various industrial applications. Overall, the research and development of magnetically responsive cellulose is an exciting and rapidly growing field with great potential for future innovations. More importantly, it is tempting to enhance the osteoinduction ability of nanocellulose-based aerogels and modulate the host immune system by gene delivery in BTE [187]. Compared with a protein molecular delivery system, gene delivery therapy, which is based on the delivery of therapeutic genes and subsequent protein expression for bone regeneration, has gained more remarkable achievements over the past few decades [188,189]. However, nanocellulose-based aerogel scaffolds for gene delivery therapy have rarely been studied, which will be one of the research directions in the next few years.

Through 3D printing, the personalized design of macroscopic structures could be effectively realized, and the properties of cellulose aerogels for specific applications can be fine-tuned and improved. The use of 3D bioprinting can provide cellular support while constructing tissues and organs, facilitating cell growth and settlement. Compared to traditional 3D printing, 3D bioprinting has the advantages of higher precision in detail, stronger biocompatibility, and better support for cells. Therefore, it has tremendous potential in the field of biomedical research and medicine. Cellulose composite materials and their derivatives can serve as suitable 3D bioink for bioprinting.

## Figures and Tables

**Table 1:** Types of crosslinking [63].

Crosslinking	Crosslinker	Advantages	Disadvantages
Physical	Hydrogen linkages, electrostatic interaction, ionic crosslinking, p–p stacking, dehydration heat treatment, and ultraviolet treatment [68,71]	Safe, cheap, small tissue response	Low degree of crosslinking, difficult to control the crosslinking reaction, time-consuming
Chemical	GA, CA, GP [73], HMDA [74], TA [75]	Forming a strong covalent bond	Cytotoxic
Enzymatic	H_2_O_2_, horseradish peroxidase, transglutaminase, tyrosinase [72]	Controlled by temperature, pH, or ionic strength	Expensive price, substrate specificity

**Table 2:** Cellulose aerogel in bone tissue engineering.

Composite	Preparation Method	Porosity or Pore Size	Mechanical Properties	Seeding Cell	Results	Year	Ref.
HA–CNC	Esterification reaction and freeze-drying	91%	Compressive strength: 41.8 MPa		Biodegradable, non-toxic, low immunogenicity, and biocompatibility flexible-shaped ability	2018	[113]
CNC	Hydrazone crosslinking and CO_2_ supercritical drying	98.8–99.3%	Young’s modulus: 25–65 KPa	Osteoblast-like Saos-2 cells	High porosity and effective bone growth promotion osteoconduction	2019	[64]
HA–BC	Freeze-drying (cryogels) and scCO_2_ drying(aerogels)	30–80 nm	Elastic modulus: 10.91 ± 3.26 G Pa, hardness of 0.37 ± 0.18 G Pa.	-	Excellent mechanical strength	2019	[102]
Gelatin–CNF	HMDA crosslinking	94–95%	35.2–54.7 KPa	L929 fibroblasts	Suitable for cell adhesion and growth	2019	[74]
	Freeze-drying	300 μm					
CS–CMC	GA crosslinking and freeze-drying	82 ± 5%	Strength: 2.51 GPa modulus: 139 MPa	MG63	The cell viability increased significantly	2019	[114]
		Mesoporous: >100 μm					
		Micropore: <50 μm					
PEGDA–CNF	SLA and freeze-drying	Average pore size: 46–69 μm	The elastic deformation was 35 KPa under 30% stress	BMSC	Suitable for cell adhesion and growth	2019	[115]
PCL–CS-cellulose acetate	Electrospinning and freeze-drying	-	The compression modulus can reach 0.31 MPa modulus of compression: 45 ± 6 Kpa	MC3T3-E1	Improve cell adhesion, infiltration, and osteogenic differentiation	2020	[100]
SF–cellulose	Chemical crosslinking and freeze-drying	-	Tensile strength: 7.73 MPa	HEK-293 T cells	Excellent mechanical strength	2021	[116]
			Strength of bending: 25.91 MPa				
CS–CNF	Freeze-drying	97.20%	Young’s modulus: 0.28 MPa	-	Excellent mechanical properties	2021	[117]
SF–n–HA–cellulose	Chemical crosslinking and freeze-drying	99.20%	Young’s modulus: 12.7–22.4 MPa	HEK-293T cells	Controllable degradation rate;	2021	[37]
					Good mineralization ability;		
PEGDA–CNF	Stereolithography and freeze-drying	Mesoporous: 400–800 um	Young’s modulus: 2.94 MPa	Mouse BMSC	Controllable pore structure	2021	[118]
		Micropore size: 20~100 µm			Adjustable Poisson’s ratio		
CS–CNC	Chemical crosslinking and CO_2_ supercritical drying	20–60 nm	Compressive strength was 0.13 MPa at 3% strain	-	Reduce the gel shrinkage	2021	[103]
BC	Freeze-drying and seeded with BMP2	Macropores: >100	-	BMSC	Excellent osteoconduction	2021	[17]
		μm, micropores: <100 μm, nanopores: <100 nm			Osteoinduction		
COL–n–HA–CNF	Thermal crosslinking	90%	The elastic modulus was (12.95 ± 4.77) MPa, and the compressibility was (0.4067 ± 0.084) MPa.	Rabbit BMSC and human vascular endothelial cells	Control releasing ability; osteogenesis and vascularization abilities.	2022	[119]
	and freeze-drying	75 ± 18 µm					
ɛ-poly-l-lysine-TEMPO CNF	Esterification, crosslinking with CA and freeze-drying	≥85.05%	Tensile strength: 22 MPa	-	Antibacterial property and degradable	2022	[120]
PEGDA/cellulose	SLA and freeze-drying	20–50 μm	0.58 ± 0.0222 MPa	BMSC	Dynamic Poisson’s ratio promotion differentiation at different stages of BMSC	2022	[121]

**Table 3:** Characteristics of esterification and etherification modification of cellulose.

Derivatization/Modification Method	Introduced Functional Groups	Advantages	Surface Property	Ref.
Esterification	-COOR	Enhanced hydrophobicity and mechanical strength. Reduced water uptake. The specific surface area of cellulose increased.	FTIR confirmed the occurrence of carboxylic esterification on hydroxyl groups. The particle size decreased by 25–35 µm DY11 compared to the original cellulose, while the adsorption amount increased by 20–30 mg/g	[178]
Etherification	-OR |	Enhanced hydrophobicity, mechanical strength, and thermal stability. Improved hydrophilicity.	The FTIR spectra results indicate the presence of carboxyl characteristic peaks. TGA analysis shows higher thermal stability.	[179]

Direct Yellow 11 (DY11) was used to evaluate cellulose accessibility through the modified Simons’ staining.

## Data Availability

Not applicable.
